# The Heparan Sulfate Proteoglycan Syndecan-1 Triggers Breast Cancer Cell-Induced Coagulability by Induced Expression of Tissue Factor

**DOI:** 10.3390/cells12060910

**Published:** 2023-03-16

**Authors:** Nourhan Hassan, Nico Bückreiß, Janes Efing, Marie Schulz-Fincke, Philipp König, Burkhard Greve, Gerd Bendas, Martin Götte

**Affiliations:** 1 Department of Gynecology and Obstetrics, Münster University Hospital, Albert-Schweitzer-Campus 1, 48149 Münster, Germany; 2 Biotechnology/Biomolecular Chemistry Program, Faculty of Science, Cairo University, Giza 12613, Egypt; 3 Pharmaceutical Department, University Bonn, An der Immenburg 4, 53225 Bonn, Germany; 4 Department of Radiotherapy-Radiooncology, Münster University Hospital, Albert-Schweitzer-Campus 1, 48149 Münster, Germany

**Keywords:** apoptosis, breast cancer, cell cycle, cell motility, heparan sulfate, platelets, signal transduction, syndecan-1, thrombin, tissue factor

## Abstract

Syndecan-1 (Sdc-1) upregulation is associated with poor prognosis in breast cancer. Sdc-1 knockdown results in reduced angiogenesis and the dysregulation of tissue factor (TF) pathway constituents. Here, we evaluate the regulatory mechanisms and functional consequences of the Sdc-1/TF-axis using Sdc-1 knockdown and overexpression approaches in MCF-7 and MDA-MB-231 breast cancer cells. Gene expression was analyzed by means of qPCR. Thrombin generation and cell migration were detected. Cell-cycle progression and apoptosis were investigated using flow cytometry. In MDA-MB-231 cells, IL6, IL8, VEGF, and IGFR-dependent signaling affected TF pathway expression depending on Sdc-1. Notably, Sdc-1 depletion and TF pathway inhibitor (TFPI) synergistically affected PTEN, MAPK, and STAT3 signaling. At the functional level, the antiproliferative and pro-apoptotic effects of TFPI depended on Sdc-1, whereas Sdc-1’s modulation of cell motility was not affected by TFPI. Sdc-1 overexpression in MCF-7 and MDA-MB-231 cells led to increased TF expression, inducing a procoagulative phenotype, as indicated by the activation of human platelets and increased thrombin formation. A novel understanding of the functional interplay between Sdc-1 and the TF pathway may be compatible with the classical co-receptor role of Sdc-1 in cytokine signaling. This opens up the possibility of a new functional understanding, with Sdc-1 fostering coagulation and platelet communication as the key to the hematogenous metastatic spread of breast cancer cells.

## 1. Introduction

Syndecan-1 (Sdc-1) is the most extensively characterized of the four members of the syndecan family of transmembrane heparan sulfate proteoglycans (HSPGs) [1]. Via its highly conserved cytoplasmic domains, Sdc-1 interacts with scaffolding proteins, providing a link to the cytoskeleton. Its extracellular domain allows for protein–protein interactions with integrins and complex formation with receptor tyrosine kinases (RTKs), enhancing signaling as a co-receptor [2]. Sdc-1 exhibits binding interactions with a plethora of growth factors, chemokines, morphogens, proteases, protease inhibitors, and matrix glycoproteins, which are mediated by its HS glycosaminoglycan chains [1,2].

Sdc-1 expression is upregulated in breast cancer, the most frequent malignancy in women. It is associated with aggressive cellular properties and poor prognosis [3]. At the cellular level, Sdc-1 modulates pro-metastatic proteolytic activity and cell motility and fosters the cancer stem cell phenotype. Its molecular mechanisms include the dysregulation of inflammatory and angiogenic cytokines, tissue inhibitors of metalloproteinases (TIMPs), matrix metalloproteinases (MMPs), and heparanase, as well as altered integrin-related, RTK, Notch, and morphogen signaling [4,5,6].

Notably, Sdc-1 not only determines tumor cell-autonomous functions, but also affects the tumor microenvironment, including the immune cell phenotype, extracellular matrix (ECM) organization by stromal fibroblasts, and tumor angiogenesis [6,7,8].

For example, Sdc-1 expression is associated with angiogenic factors in ductal breast carcinoma in situ, its interaction with αvβ3 and αvβ5 integrins regulated angiogenesis in a murine breast cancer model, and Sdc-1 expression by stromal fibroblasts enhances tumor growth and angiogenesis [6,9,10]. In vitro, Sdc-1 downregulation decreased the expression and secretion of angiogenic factors in breast cancer cells [11]. Utilizing a 3D co-culture model of breast cancer cells and human umbilical vein endothelial cells, we demonstrated that Sdc-1 depletion in tumor cells decreased endothelial tubule network formation and decreased the expression and secretion of vascular endothelial growth factor (VEGF) and several constituents of the tissue factor (TF) pathway [11]. However, its functional relevance and the mechanisms beyond angiogenesis have not yet been evaluated.

TF, also known as factor III (F3) or CD142, is considered a key factor fostering the hematogenous metastasis of cancer cells [12]. Many tumor cells express TF and thus activate the plasmatic coagulation cascade, providing local thrombin formation when entering the blood system. Thrombin, in turn, is a highly potent mediator when activating its protease-activated receptor (PAR) pathways in tumor cells; endothelial cells; and, most importantly, platelets. The interaction of tumor cells with platelets is likely the most decisive step in hematogenous cancer cell dissemination [13]. Immediately after arriving in the blood, tumor cells activate platelets via the TF/thrombin pathway, resulting in the formation of a tight platelet cloak around the tumor cells, protecting them from shear stress and immune surveillance, e.g., detrimental NK cell assaults [14]. Platelets confer multiple further survival advantages on tumor cells by affecting the epithelial-to-mesenchymal transition (EMT) and increasing their motility and invasiveness. Therefore, targeting of the tumor cell-induced platelet activation with anticoagulants is considered a promising approach to interfere with metastatic spread [15]. However, the use of Sdc-1 has not yet been addressed from this perspective. Although both Sdc-1 and TF have been associated separately with malignancy and coagulability, the identification of an axis of Sdc-1, TF, and coagulation [11] is novel and of potential relevance for understanding the pathogenesis of breast cancer. However, the mode of action and regulation of this axis has been only poorly defined and remains to be confirmed. Therefore, in this study, we used Sdc-1 knockdown and overexpression approaches in breast cancer cell lines in vitro to elucidate the regulatory and signaling pathways associated with the Sdc-1/TF/coagulation axis, to study its impact on further cellular properties including cell cycle progression and cell motility, and to analyze its consequences for platelet activation.

## 2. Materials and Methods

### 2.1. Cell Culture

MCF-7 and MDA-MB-231 human breast cancer cell lines were purchased from ATCC/LGC Promochem (Wesel, Germany). Cells were cultured as described previously [16]. Regarding Sdc-1 overexpression, cells were stably transfected with a pcDNA3.1 control plasmid (Invitrogen, Karlsruhe, Germany) or a plasmid enabling the overexpression of wild-type Sdc-1 (Sdc-1-OV) and a constitutively shed variant (392-Sdc-1-OV) of murine Sdc-1 in the vector pReceiver-M02. Using 800 µg/mL of G418, stable clones were selected and characterized [5].

### 2.2. siRNA Transfection

MDA-MB-231 cells were cultured in six-well plates in growth media at a density of 350,000 cells/well. Then, 24 h after seeding, they were transfected with 20 nM pre-validated siRNA (Ambion life technologies, Cambridgeshire, UK) targeting exon 2 of Sdc-1 (NM_002997.4) to achieve the knockdown of Sdc-1 expression or a Silencer^TM^ Select negative control siRNA (Ambion) using Dharmafect reagent (DharmaconTM, Lafayette, CO, USA), according to the supplier’s protocols and as described previously, using a ratio of 2 µL/mL Dharmacon reagent and 20 pmol/mL siRNA [3,4].

### 2.3. Cytokine, Inhibitor and TFPI Treatment of Breast Cancer Cells

After transfection, cells were either treated with different concentrations of cytokines or inhibitors for 48 h or left untreated (control) (Table S1). TFPI (Merck KGaA, Darmstadt, Germany) was used at a final concentration of 50 ng/mL. All treatments were applied under serum starvation conditions.

### 2.4. Quantitative Real-Time PCR

Total RNA extraction, cDNA synthesis, and quantitative real-time PCR were performed as previously described [11]. Relative gene expression levels were assessed using the 2-ΔΔCt method after normalization to β-actin expression, which served as an internal control. Specific product amplification was confirmed by means of melting curve analysis. Corresponding primer sequences were validated using NCBI BLAST and are listed in Supplementary Table S2.

### 2.5. Protein Extraction and Western Blot

Transiently transfected cells were treated with TFPI for 48 h. The preparation of total cell lysates, gel electrophoresis, and Western blotting were performed as previously described [5,17]. Primary and secondary antibodies are listed in Supplementary Table S3.

### 2.6. MTT Metabolic Cell Viability Assay

Sdc-1-depleted cells or controls (5 × 10^3^) treated with TFPI were plated in 96-well plates with DMEM medium (without phenol red) (Gibco^®^, Dreieich, Germany) with FCS and incubated for 96 h. Afterwards, the estimation of the cell proliferation was performed exactly as described previously [5]. The number of proliferated cells in the control group was defined as 100%. Three independent sets of triplicate experiments were used to derive the results. *N*,*N*-Dimethyl formamide, MTT, and SDS were obtained from Sigma-Aldrich Chemie GmbH, Taufkirchen, Germany.

### 2.7. Cell Cycle Analysis

Cells were washed twice with ice-cold PBS and harvested with 2 mM EDTA (AppliChem, Darmstadt, Germany), which was applied for 13 min at 37 °C, and subsequently stained with 4,6-diamidino-2-phenylindole (DAPI) by resuspending the cell pellet in 1 mL of DAPI solution (Cytecs, Münster, Germany). After 5 min of incubation at room temperature, cells were analyzed using a CyFlow space flow cytometer (PARTEC, Münster, Germany) and a Guava easyCyte 3 HT reader (Merck Millipore; Billerica, MA, USA). Excitation was carried out with a 375 nm UV laser and fluorescence emissions were measured at 455 nm in FL4. For data analysis, FloMax software (Quantum Analysis, Münster, Germany) and GuavaSoft InCyte (v.3.3) (Luminex, Austin, TX, USA) were used.

### 2.8. Apoptosis Assay

To measure the proportion of apoptotic cells, the detached cells in all conditions were stained with the FITC Annexin V Apoptosis Detection Kit (BD Pharmingen, Franklin Lakes, NJ, USA) as previously described [17]. Annexin V-FITC-labeled cells were detected using the CyFlow space device (Sysmex PARTEC GmbH). Data analysis was performed using FloMax software (Partec).

### 2.9. Scratch Migration Assay

Four hundred thousand cells/well were seeded in six-well plates and were transfected with Sdc-1 siRNA and negative control siRNA for 24 h. After transfection, treatment with 50 ng/mL TFPI was performed for 24 h. Forty-eight hours after transfection, the confluent cell layers were disrupted by scraping once horizontally and once vertically with a pipette tip. The closing of the resulting cell-depleted gap was monitored by means of Nomarski contrast light microscopy and documented with a Zeiss Axiophot camera (Zeiss, Jena, Germany) directly after 0, 24, and 48 h. Cell-free areas were quantified using NIH ImageJ software (NIH, Bethesda, MA, USA).

### 2.10. Migration Assay

Sdc-1 depleted MDA-MB-231 cells and controls with/without TFPI treatment were diluted to 50,000 cells/mL in DMEM without FCS. Then, migration chambers (Sigma-Aldrich Chemie GmbH, Taufkirchen, Germany) were supplied with 500 μL (25,000 cells). The migration was triggered by adding 750 μL of DMEM with 5% FCS to the lower wells. After 24 h, the cells were fixed in methanol for 6 min, washed with PBS for 1 min, and stained with 1% toluidine blue in BORAX. Cells on top of the chamber were removed with “cotton-wool” sticks and cells on the bottom membrane were analyzed by means of microscopy.

### 2.11. Light Transmission Aggregometry

To evaluate breast cancer cell-induced platelet aggregation, light transmission aggregometry (LTA) was applied using an APACT 4004 semi-automatic platelet aggregometer (Haemochrom Diagnostica, Essen, Germany) as described previously [18]. Therefore, human platelet concentrates were used, which were centrifuged at 670× *g* for 10 min at room temperature and residual plasma was discarded. The pelleted platelets were resuspended in a platelet buffer to a final concentration of 4 × 10^8^ platelets/mL to obtain platelet-rich buffer (PRBu) [19]. Immediately before measurement, PRBu was recalcified to a 2.5 mM physiological concentration by adding a 0.5 M CaCl_2_ solution. Subsequently, PRBu was aliquoted into cuvettes, and aggregation was induced by adding 1 × 10^5^ tumor cells/mL under permanent stirring (1000 rpm) at 37 °C. Platelet-poor buffer was used as a positive control (100% aggregation) and PRBu was used as a negative control (0% aggregation).

### 2.12. Thrombin Generation Assay

Cancer-cell-induced endogenous thrombin generation potential (ETP) in PRBu was evaluated using a TECHNOTHROMBIN^®^ assay kit (Technoclone, Vienna, Austria), which is based on thrombin-specific cleavage of a fluorogenic substrate, Z-Gly-Gly-Arg-AMC. By adding plasma, the platelet concentrates were diluted to a concentration of 4 × 10^8^ platelets/mL (PRP). Since thrombin generation can be initiated by both the extrinsic (factor VII) and intrinsic (factor XII) pathways [20], factor XIIa inhibitor (corn trypsin inhibitor, Santa Cruz Biotechnology, Heidelberg, Germany) (50 µg/mL PRP) was added. Afterwards, PRP was transferred to a black 96-well plate (ThermoFisher Scientific Inc., Waltham, MA, USA) (40 µL/well) and treated with DPBS as a negative control, recombinant TF as a positive control, or the respective breast cancer cells (1 × 10^5^ tumor cells/mL). Shortly after adding the fluorogenic substrate, the kinetics of thrombin formation was continuously measured using a Tecan Spark microplate reader (Tecan Group, Männedorf, Switzerland). Technoclone evaluation software was used to convert the fluorescence signals to thrombin concentrations.

### 2.13. Flow Cytometric Analysis of TF Expression

The TF expression of tumor cells was determined by means of flow cytometry. Therefore, cells were incubated with a mouse anti-TF antibody (ThermoFisher Scientific Inc.) for 45 min at a concentration of 2.5 µg/1 × 10^6^ tumor cells in a 96-well plate. Before labeling cells with a FITC-conjugated secondary anti-mouse antibody (BD Biosciences, Heidelberg, Germany) (1 µg/well) for a further 45 min, cells were washed once. A Guava easyCyte 3 HT reader (Merck Millipore) and GuavaSoft InCyte (v.3.3) were used for analysis.

### 2.14. STRING Protein–Protein Interaction Analysis

The online STRING analysis tool (https://string-db.org/ (accessed on 1 September 2022)) was used to develop an in-silico protein interaction network for Sdc-1, TF, and TFPI in the presence of interleukin (IL) 6, IL8, VEGF, and IGF-1R. Interactions were predicted with a high confidence threshold of 0.700. The STRING tool additionally uses the databases GO, Pfam (Protein families), and KEGG, and performs multiple testing corrections separately within each functional classification framework to predict protein–protein interaction (PPI) networks [21].

### 2.15. Statistical Analysis

Statistical analysis was performed using 95% confidence intervals (CI), determined by calculating the arithmetic mean values and variance (standard deviation, SD) of at least three independent biological replicates. Student’s *t*-test (*t*-test, Mann–Whitney) was applied when comparing two groups and one-way ANOVA with Turkey’s multiple comparisons test was used when comparing more than two groups with each other. Prism software (GraphPad, San Diego, CA, USA) was used for statistical analysis and graphical representation, considering *p*-values < 0.05 to be statistically significant.

## 3. Results

### 3.1. Sdc-1 Knockdown Modulates Cytokine-Dependent Tissue Factor (TF) Pathway Gene Expression in MDA-MB-231 Breast Cancer Cells

We first addressed the question of which Sdc-1-dependent pathway was responsible for the previously observed dysregulation of the TF pathway in Sdc-1-depleted breast cancer cells [11]. Therefore, we suppressed Sdc-1 expression using siRNA knockdown (KD) in the triple-negative breast cancer cell line MDA-MB-231 to assess its effect on the TF pathway in conjunction with various cytokine treatments, referring to the role of Sdc-1 as a co-receptor for these pathways [2]. RT-qPCR was used to confirm the silencing of Sdc-1 (Figure S1A). Sdc-1-siRNA-KD has been documented to be stable for at least 10 days, causing a decrease in the protein level [5].

**Figure 1:**
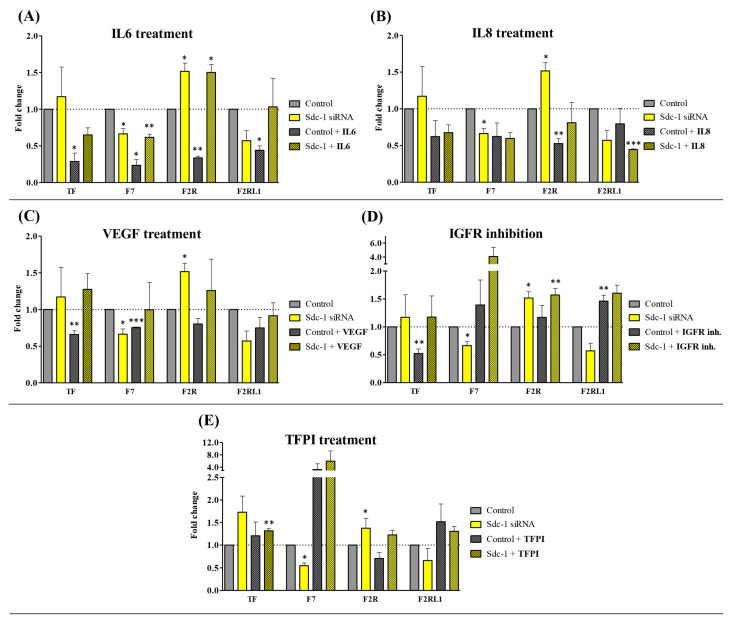
Sdc-1-KD modulates cytokine-dependent tissue factor (TF) pathway gene expression in MDA-MB-231 breast cancer cells. The expression of TF pathway-associated genes was analyzed post-transfection and 24 h after treatment with exogenous (**A**) IL6 (100 ng/mL), (**B**) IL8 (100 ng/mL), and (**C**) VEGF (20 ng/mL) and with (**D**) the inhibition of IGFR with AG-1024 (20 μM) and (**E**) TFPI (50 ng/mL) by means of qPCR, normalized to β-actin expression and shown as a fold change (n = 3). Data are presented as bar plots and means are represented with lines. Bars with asterisks represent comparisons with statistically significant differences (* *p* < 0.05, ** *p* < 0.01, *** *p* < 0.001).

To study the impact of Sdc-1-KD on the TF pathway, we analyzed the mRNA expression of TF; coagulation factor VII (F7); coagulation factor II thrombin receptor (F2R), also known as protease-activated receptor (PAR)-1; and coagulation factor II (thrombin) receptor-like 1 (F2RL1), also known as PAR-2, by means of qPCR [11]. Seventy-two hours after Sdc-1 downregulation, significantly diminished F7 expression and significant upregulation in the expression of F2R were observed compared to control cells (Figure 1), whereas TF and F2RL1 were not affected. To characterize which molecular pathways may be involved in Sdc-1-dependent TF pathway constituent expression, we stimulated cells with exogenous IL6, IL8, or VEGF, or treated them with TFPI or IGFR inhibitors, respectively. Treatment of control MDA-MB-231 cells with IL6 resulted in significant downregulations of TF (0.28, *p* < 0.05), F7 (0.23, *p* < 0.05), F2R (0.33, *p* < 0.01), and F2RL1 (0.44, *p* < 0.05) (Figure 1A). Sdc-1-KD reversed this effect, leading to the upregulation of TF and F7 partially to control levels (F2RL1) or even exceeding these levels (F2R), suggesting that Sdc-1 is required for proper IL6 signaling in this context. With respect to IL8 treatment, only F2R was significantly downregulated in control cells. This effect could be abolished by Sdc-1-KD (Figure 1B). Sdc-1-dependent downregulation of F2RL1 expression was further enhanced by IL8 treatment, suggesting complex regulatory interactions (Figure 1B). Treatment of MDA-MB-231 cells with VEGF resulted in a significant downregulation of TF (0.65, *p* < 0.01) and F7 (0.75, *p* < 0.01), which was reversed by Sdc-1-KD, which is again compatible with a potential role as a signaling coreceptor (Figure 1C). IGFR inhibition resulted in the downregulation of TF and the upregulation of F2RL1 (1.46, *p* < 0.01) in control cells, which was abolished by Sdc-1-KD in the case of TF (Figure 1D). The application of TFPI only resulted in a slight upregulation of TF in Sdc-1-depleted cells (Figure 1E). Overall, these data suggest that cytokine-dependent regulation of the TF pathway in MDA-MB-231 cells is partially dependent on the presence of Sdc-1.

### 3.2. Sdc-1-KD in MDA-MB-231 Cells Is Associated with a Differential Kinase Activation Profile of Downstream Targets of TF Signaling

**Figure 2:**
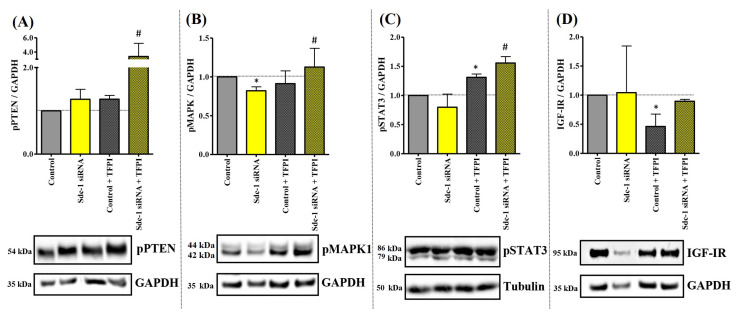
Sdc-1-depletion in MDA-MB-231 is associated with a differential kinase activation profile of different downstream protein targets of TF signaling. Phosphorylated forms of (**A**) PTEN (Ser380), (**B**) p44/42 MAPK (Erk1/2) (Thr202/Tyr204), and (**C**) STAT3 (Tyr705), as well as (**D**) the expression of IGF-1R, were analyzed after Sdc-1-KD and 48 h of 50 μg/mL TFPI treatment by means of Western blotting. Data were normalized to GAPDH levels (n = 3). Representative bands and quantitative analysis via densitometric scanning are shown. Data represent the mean ± SEM. Asterisks on the top of the bars indicate significant differences between controls and all the conditions. The line sover the bars indicate a significant difference (# *p* < 0.08, * *p* < 0.05).

To understand which molecular signals are triggered in MDA-MB-231 cells after Sdc-1-KD, we analyzed the activation state of mitogen-activated protein kinase (MAPK), phosphatase and tensin homolog (PTEN), signal transducer and activator of transcription 3 (STAT3), nuclear factor kappa B (NFkB), and insulin-like growth factor 1 receptor (IGF-1R) signaling by means of Western blot analysis. The contribution of the TF pathway was studied using TFPI. Under basal conditions, Sdc-1-KD resulted in a significant reduction in MAPK signaling (Figure 2A). Notably, the combination of Sdc-1-KD and TFPI treatment resulted in an increase in PTEN, MAPK1, and STAT3 activation (Figure 2B–D). In control cells, TFPI treatment resulted in a significant increase in STAT3 activation and decreased IGF-1R phosphorylation (Figure 2C,D), respectively. No differences were found regarding NFkB phosphorylation (results not shown). STAT3 phosphorylation was enhanced when cells were treated with TFPI alone or when combined with Sdc-1 depletion (Figure 2C). These results indicate that the differential phosphorylation of these pro-tumoral pathways at least partially depends on an interplay between TFPI and the presence of Sdc-1.

To study whether downstream pathways were modulated by Sdc-1-KD, we analyzed the expression of EGFR, MAPK1, NFkB, endothelin-1 (EDN1), and insulin-like growth factor-binding protein 1 (IGFBP1) by means of qPCR 72 h post-transfection. Furthermore, we analyzed their expression under exogenous IL8, VEGF, and TFPI treatments to evaluate whether Sdc-1-KD affected the different modulations of its receptors (Supplementary Figure S1B–E). Only EDN1 was significantly upregulated in Sdc-1-depleted, VEGF-treated, and IL8-stimulated MDA-MB-231 cells (1.54, *p* < 0.01; 1.877, *p* < 0.01), respectively (Figure S1B,C). Sdc-1-KD led to a significant decrease in IL8 expression irrespective of TFPI treatment (0.32, *p* < 0.01) (Figure S1E), whereas the significant decrease in IL6 (0.311, *p* < 0.01) and the significant upregulation of survivin expression (2.31, *p* < 0.01) in TFPI-treated controls were abolished by Sdc-1-KD (Figure S1E).

### 3.3. TFPI Treatment Exerts Different Effects on Cell Proliferation, Cell Cycle, and Invasiveness with Respect to Sdc-1-KD in MDA-MB-231 Cells

**Figure 3:**
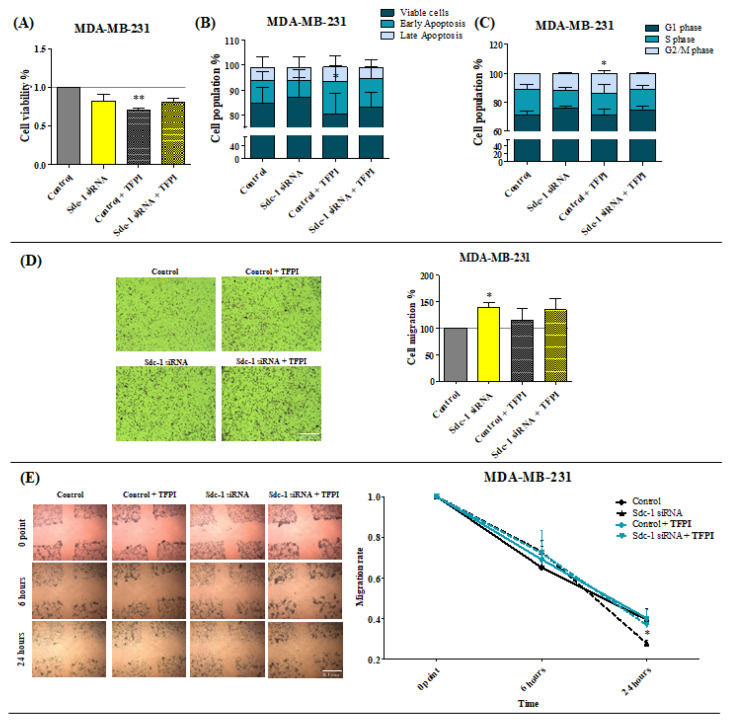
Treatment with TFPI decreases the viability and invasive phenotype of MDA-MB-231 cells. (**A**) The MTT assay revealed a significant effect of TFPI treatment on cell viability (n = 3). (**B**) Early apoptotic MDA-MB-231 cells were found to be increased with respect to TFPI (50 μg/mL) treatment. Apoptosis was determined after Sdc-1-depletion (24 h) and 48 h after 50 μg/mL of TFPI treatment with annexin V and PI staining and by means of flow cytometry, and the percentage of viable (annexin V−/PI−), early apoptotic (annexin V+/PI−), and late apoptotic (annexin V+/PI+) cells are plotted as a percentage of the cell population (n = 3, MDA-MB-231). (**C**) TFPI treatment promotes cell cycle progression in MDA-MB-231 cells. Cell cycle phase composition was measured by means of DAPI staining and flow cytometry after Sdc-1-depletion and 48 hours of 50 μg/mL TFPI treatment (n = 3, MDA-MB-231). (**D**,**E**) Sdc-1-KD increased the migratory characteristics of MDA-MB-231 cells, as determined using transwell migration and wound-healing (scratch) assays. The **left** panel shows representative images of both assays (10× magnification) and the **right** panel shows the percentage of cell migration for the migration assay and the rate of migration for the scratch assay. Scale bars = 100 μm. Data represent mean ± SEM. Asterisks on the top of the bars indicate significant differences between the control and all the conditions. Lines over the bars indicate significant differences (* *p* < 0.05, ** *p* < 0.01).

Cell proliferation, apoptosis, cell cycle progression, and invasive growth are relevant processes involved in cancer development [22]. We previously demonstrated the impact of Sdc-1 KD on these processes in MDA-MB-231 breast cancer cells [4,11,17]. To define the contribution of Sdc-1-dependent TF pathway regulation to these pathogenetic routes, we analyzed these processes in MDA-MB-231 cells subjected to Sdc-1-KD and/or TFPI treatment, respectively (Figure 3). MTT assays revealed a significant decrease in cell viability upon TFPI treatment (0.703, *p* < 0.01) of control cells, which was not seen upon Sdc-1-KD (Figure 3A). A significant increase in the number of early (annexin V+/PI−) apoptotic cells, with an associated decrease in viable cells, was observed after the treatment with TFPI, with respect to control cells (Figure 3B). Again, this effect was abolished by Sdc-1-KD. Evaluating whether Sdc-1-KD and/or TFPI treatment had an impact on cell-cycle progression, we observed that MDA-MB-231 cells had a higher proportion of cells in the G2/M phase than control cells upon TFPI treatment. However, Sdc-1-depleted MDA-MB-231 cells did not show changes in their cell-cycle phase composition (Figure 3C).

We next investigated whether Sdc-1-KD or TFPI affected MDA-MB-231 cell motility. Notably, Sdc-1-KD increased the invasive potential by 38% (*p* < 0.05) in a transwell migration assay, compared to the control (Figure 3D). In contrast, TFPI treatment did not significantly modulate the invasion characteristics of MDA-MB-231 cells. In a complementary wound-healing assay, wound closure was facilitated by Sdc-1-KD compared to controls after 24 h (Figure 3E). Again, TFPI treatment did not affect this Sdc-1-dependent process.

These data suggest that Sdc-1 expression is required for TFPI-mediated effects on MDA-MB-231 cell viability, apoptosis, and cell cycle progression, whereas the cell migration-dependent effect of Sdc-1 depletion is not affected by TFPI.

### 3.4. The Expression of TF and TFPI in Different Breast Cancer Cell Lines Represents Different Molecular Classifications

**Figure 4:**
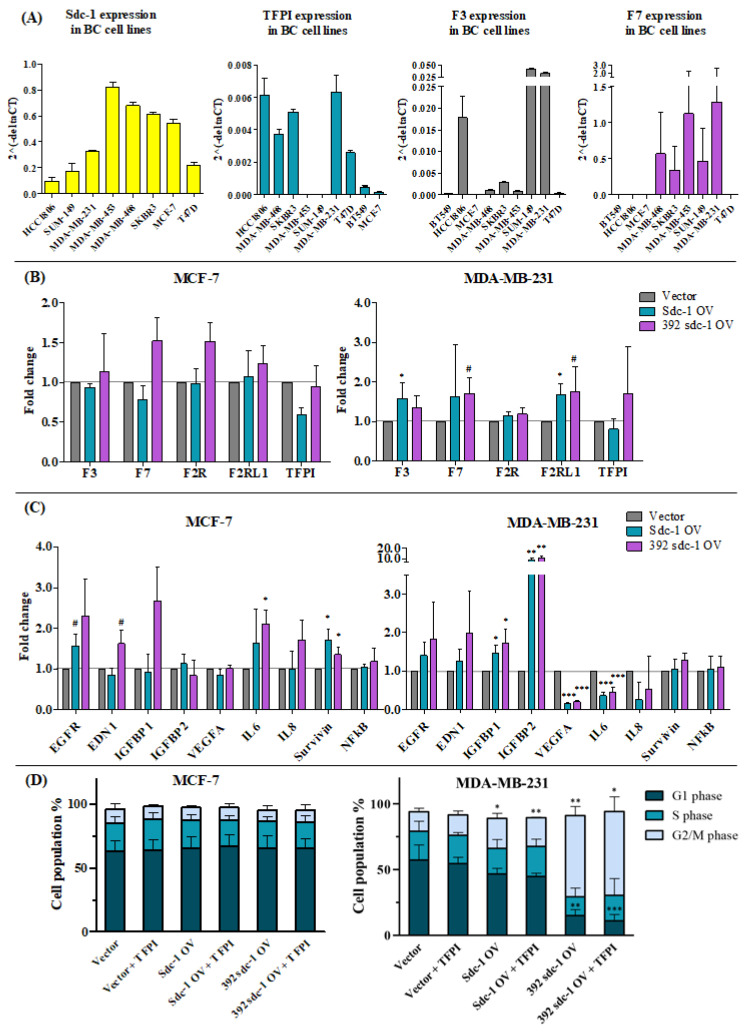
Sdc-1 overexpression has a differential effect on the tissue factor pathway (TF, F7, F2R, F2RL1, and TFPI) and cell-cycle progression in MCF-7 and MDA-MB-231 breast cancer cells. (**A**) Relative expression of Sdc-1, TFPI, TF, and F7 was quantified via qPCR in nine breast cancer cell lines, representative of the basal (MDA-MB-231, -468, -453, and BT549), luminal (T47D, SUM-149, and MCF-7 (WT status for p53)), and HER2-enriched (SKBR3 and BT-474) subtypes. Individual experiments were normalized against β-ACTIN and the relative expression was represented by means of 2-ΔCt. (**B**,**C**) The expression of tissue factor pathway-associated genes was analyzed in Sdc-1-OV and 392-Sdc-1-OV breast cancer cells by means of qPCR, normalized to β-actin expression, and shown as a fold change (n = 3). The **left** and **right** panels represent the MCF-7 and MDA-MB-231 cell lines, respectively. Data are presented as bar plots and means are represented with lines. Bars with asterisks represent comparisons with statistically significant differences (# *p* < 0.08, * *p* < 0.05, ** *p* < 0.01, *** *p* < 0.001). (**D**) The cell-cycle phase composition of Sdc-1-overexpressing MCF-7 and MDA-MB-231 cells was measured via DAPI staining and flow cytometry after 48 h of 50 μg/mL TFPI treatment (n = 4). Data are presented as bar plots and means are represented with lines. Bars with asterisks represent comparisons with statistically significant differences (* *p* < 0.05, ** *p* < 0.01, *** *p* < 0.001).

To study the impact of Sdc-1 overexpression on the TF pathway, we first performed qPCR pre-screening of nine breast cancer cell lines of the following subtypes [23]. Basal: MDA-MB-231, MDA-MB-468, BT549, HCC1806, and MDA-MB-453. Luminal: T47D, SUM-149, and MCF-7 (WT status for p53). HER2-enriched: SKBR3 and BT-474. Syndecan-1 expression was variable, being lower in HCC1806, SUM-149, T47D, and MDA-MB-231 cells compared to its expression in MCF-7, SKBR3, MDA-MB-468, and MDA-MB-453 cells. We previously noted that syndecan-1 protein expression was not detectable in BT549 cells (N.A. Espinoza-Sanchez, unpublished). Some cell lines (HCC-1806, MDA-MB-231, MDA-MB-468, SKBR3, and T47D) displayed strong TFPI expression, whereas others had much lower mRNA levels or failed to show this (Figure 4A, left). HCC-1806, MDA-MB-231, and SUM-149 cells showed higher TF expression than the other cell lines (Figure 4A, middle). The level of F7 expression was highest in the MDA-MB-453 and MDA-MB-231 cells compared to MDA-MB-468, SKBR3, and SUM-149 cells, which expressed much lower levels of F7 (Figure 4A, right). Taking these findings into account, we permanently overexpressed Sdc-1 and its soluble ectodomain in the luminal cell line MCF-7, as well as the basal cell line MDA-MB-231, which showed consistently large differences in TFPI, TF, and F7 expression within our cell line panel. Moreover, both cell lines represented distinct subtypes of breast cancer (luminal vs. basal), different stages of dedifferentiation (epithelial vs. mesenchymal morphology), and distinct levels of syndecan-1 expression and were thus considered suitable for evaluating the syndecan-1-dependent functions of the tissue factor pathway in different subtypes of breast cancer.

### 3.5. Sdc-1 Overexpression Affects Tissue Factor Pathway-Related Genes and Their Downstream Signaling in MCF-7 and MDA-MB-231 Cells

To complement our studies on Sdc1-depleted cells, we stably transfected MCF-7 and MDA-MB-231 cells with two different Sdc-1 DNA constructs and a control vector in order to better understand the functions of the wild-type (Sdc-1-OV) and soluble Sdc-1 ectodomain (392-Sdc-1-OV) in the modulation of the TF pathway (Figure 4B,C). Highly homologous murine constructs were used [5] and Sdc-1 overexpression was confirmed using qPCR (Figure S1F).

We first assessed the expression levels of TF pathway-related genes in the Sdc-1-OV and 392-Sdc-1-OV cells, including TF, F7, F2R, F2RL1, and TFPI, by means of qPCR. No significant alteration in gene expression was detected in MCF-7 cells between the different transfectants (Figure 4B, left). Regarding MDA-MB-231 cells, significant increases in TF (1.57, *p* < 0.05) and F2RL1 (1.66, *p* < 0.05) expression were observed in Sdc-1-OV cells, whereas trends of F7 (1.7, *p* < 0.08) and F2RL1 (1.75, *p* < 0.08) upregulation were observed in 392-Sdc-1-OV cells (Figure 4B, right).

We next evaluated the expression of downstream targets involved in TF pathway modulation, including EGFR, EDN1, IGFBP1, IGFBP2, VEGF, IL6, IL8, survivin, and NFkB. A significant increase was observed for survivin (1.71, *p* < 0.05), along with a trend of EGFR (1.57, *p* < 0.08) expression in Sdc-1-OV MCF-7 cells. IL6 (2.1, *p* < 0.05), survivin (1.35, *p* < 0.05), and EDN1 (trend) were upregulated in 392-Sdc-1-OV MCF-7 cells (Figure 4C, left). Surprisingly, in MDA-MB-231 cells, different pathways were affected by Sdc-1 overexpression. A significant increase in the expression of IGFBP2 (8.68, *p* < 0.01; 11.27, *p* < 0.01) and, to a lesser extent, in that of IGFBP1 (1.45, *p* < 0.05; 1.71, *p* < 0.05) was noted upon Sdc-1 and soluble Sdc-1 overexpression, respectively, compared to the vector control cells. Moreover, a significant decrease was observed in VEGF (0.164, *p* < 0.001; 0.199, *p* < 0.001) and IL6 (0.361, *p* < 0.001; 0.451, *p* < 0.001) in Sdc-1-OV and 392-Sdc-1-OV cells, respectively (Figure 4C, right).

Since previous studies using the overexpression of murine Sdc-1 in human breast cancer cells provided insights into its role in cell-cycle progression [24], we performed cell cycle analysis for Sdc-1-overexpressing cells with respect to TFPI treatment. Athough there was no impact of either Sdc-1 overexpression or TFPI treatment on the cell cycle in MCF-7 cells (Figure 4D, left), the overexpression of soluble 392-Sdc-1 in MDA-MB-231 cells led to a strong accumulation in the G2/M phase, causing cell cycle arrest in this phase. A slight significant increase in the cell percentage in the G2/M phase was also revealed in MDA-MB-231-Sdc-1-OV cells (Figure 4D, right).

### 3.6. Sdc-1 Overexpression Influences the Protein Expression of TF, Platelet Aggregation, and Thrombin Generation in MCF-7 and MDA-MB-231 Cells

**Figure 5:**
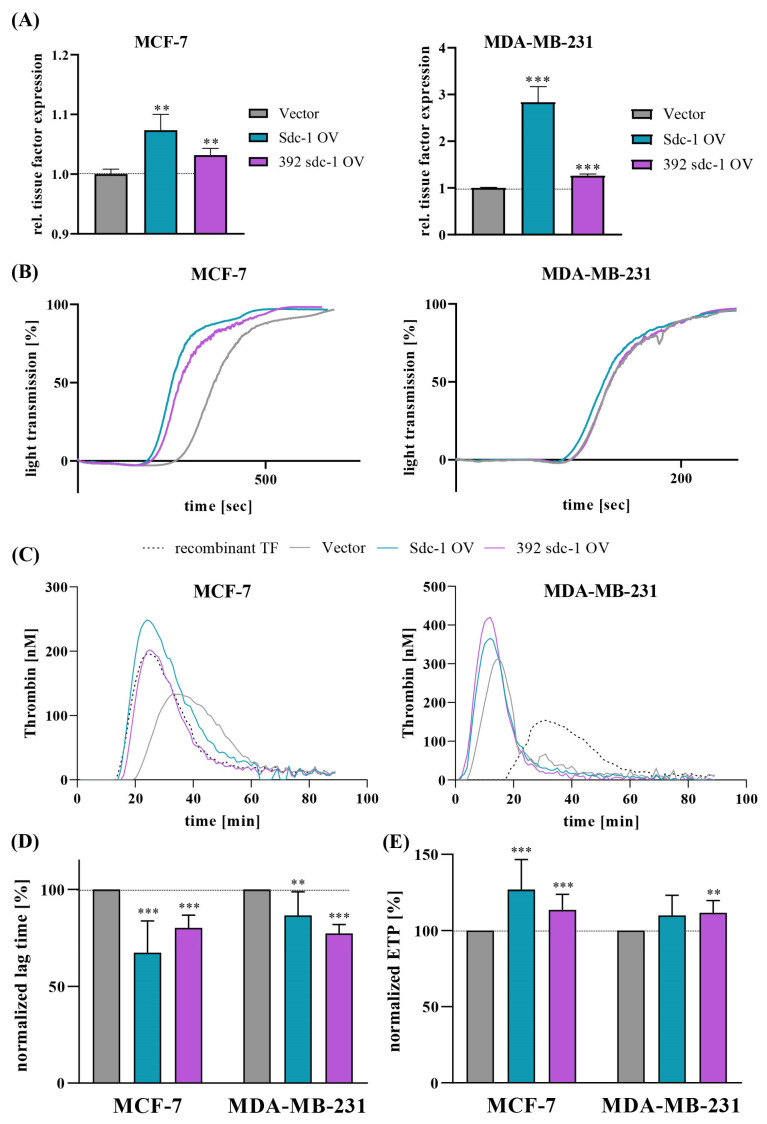
Impact of increased Sdc-1 expression on breast cancer-cell-induced platelet activation and coagulability. In all figure panels, control vector cells are displayed in gray, Sdc-1 OV in cyan, and 392 Sdc-1 OV in violet. (**A**) Flow cytometric detection results of TF expression on breast cancer cells indicated that the upregulation of Sdc-1 was associated with increased TF levels in MCF-7 cells (**left** diagram) and MDA-MB-231 cells (**right**). (**B**) Representative curves of light transmission following platelet aggregation indicated that Sdc-1 overexpressing MCF-7 cells (**left**) and MDA-MB-231 cells (**right**) tended to induce faster platelet aggregation in comparison to vector controls (n = 4). (**C**,**D**) Platelet-mediated thrombin generation induced by Sdc-1-overexpressing MCF-7 and MDA-MB-231 cells compared to vector control cells. (**C**) Representative kinetic curves of thrombin formation mediated by Sdc-1-overexpressing MCF-7 cells (**left**) and MDA-MB-231 cells (**right**) displayed a higher endogenous thrombin generation potential (ETP) with respect to increased Sdc-1 levels, confirmed by analyzing the normalized lag time, which was significantly reduced in Sdc-1-overexpressing cells (**D**), whereas ETP was significantly increased (n = 5) (**E**). Panels (**A**,**D**,**E**): Data are presented as bar plots and means are represented with lines. Bars with asterisks represent comparisons with statistically significant differences (** *p* < 0.01, *** *p* < 0.001).

To pursue the functional consequences of upregulating Sdc-1 on the postulated Sdc-1/TF axis, we first investigated whether Sdc-1 overexpression was associated with TF upregulation at the protein level. Flow cytometry data indicated that overexpression of the Sdc-1 and Sdc-1 ectodomain in MCF-7 cells was associated with a significantly higher TF expression compared to the vector cells (Figure 5A). This relationship becomes even more clear in MDA-MB-231 cells, which are known to express much higher basal levels of TF compared to MCF-7 cells [15,19]. Sdc-1 overexpression in these cells resulted in a highly significant, roughly threefold increase in TF expression at the cell surface. Although the increase was less dominant in the 392-Sdc-1-OV clones of MDA-MB-231 cells, a significant relationship between Sdc-1 and TF was confirmed at protein levels in both cell lines.

TF expression by tumor cells is a key factor in inducing the external coagulation pathway and triggers the activation of platelets in these circumstances. Platelet aggregation is a clinically established parameter that has been adapted experimentally to investigate tumor cell-induced platelet activation based on changes in light transmission [18]. Both Sdc-1-overexpressing MCF-7 clones induced enhanced platelet aggregation compared to vector controls (Figure 5B), indicated by a strongly diminished lag time until reaching full platelet aggregation. The reduction in lag time by Sdc-1-OV was more pronounced than that observed for the 392-Sdc-1-OV cells, which was in good agreement with previous flow cytometric studies (Figure 5A). Due to the much higher basal level of TF in MDA-MB-231 cells, they displayed much more rapid platelet aggregation, indicated by the bisected lag time compared to that of the MCF-7 cells. However, even in these cells, the Sdc-1-OV clones showed a slight trend towards the induction of faster aggregation. However, 392-Sdc-1-OV in MDA-MB-231 cells did not result in any changes in tumor-cell-induced platelet aggregation (TCIPA), which may be associated with already strong vector cell-mediated platelet activation and the subsequent limitations of the assay system.

TCIPA is mainly initialized by thrombin [25] via a TF coagulation pathway. Following the assumption that Sdc-1-OV cells induce stronger thrombin formation in contact with platelets, we performed a thrombin generation assay. In both MCF-7 and MDA-MB-231 cells, the Sdc-1-OV cell clones displayed faster and more intensive thrombin formation, as indicated in the representative fluorogenic thrombin formation kinetics (Figure 5C). Focusing on the more rapid thrombin formation, the significantly reduced lag times (Figure 5D) and the higher concentrations of generated thrombin, expressed as covered areas (Figure 5E), strongly indicated an increased endogenous thrombin generation potential and consequently improved coagulability mediated by cells upon Sdc-1 upregulation. These findings confirm the functional consequences of the Sdc-1/TF axis with respect to the triggering of procoagulative and thus prometastatic activities.

### 3.7. Sdc-1 Is Indirectly Linked to the TF Pathway via In Silico Interaction as a Coreceptor with Various Growth Factors, Cytokines, and Chemokines

**Figure 6:**
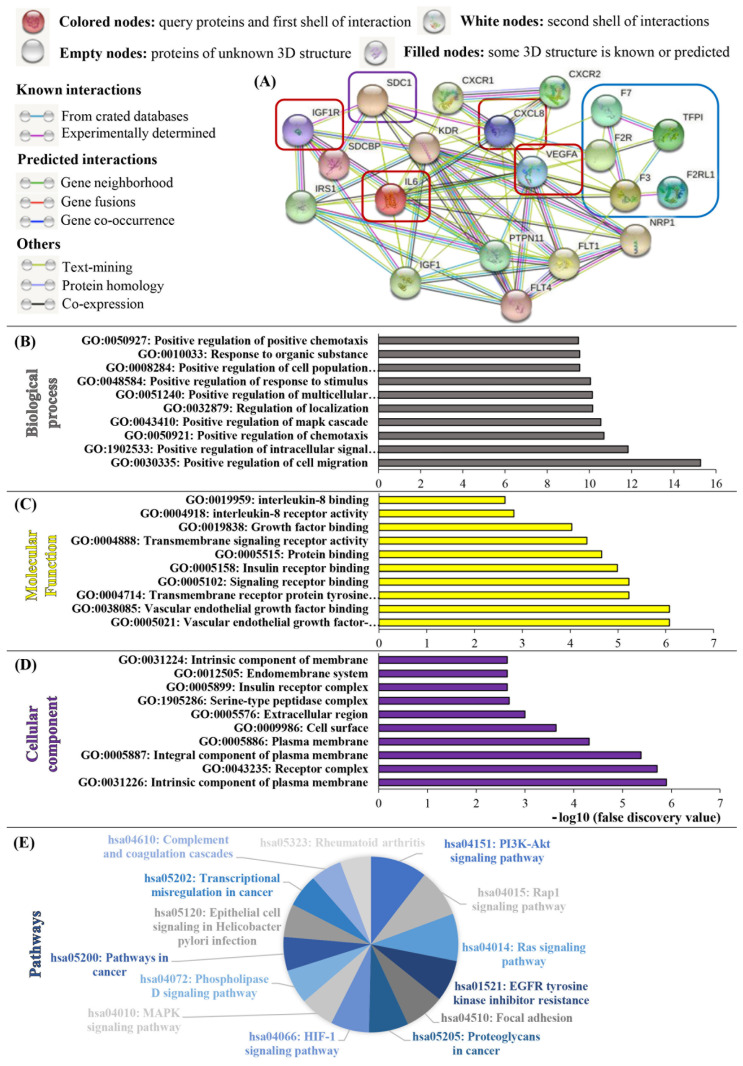
STRING analysis, revealing interactions between Sdc-1, the TF pathway, and cancer-related processes. Using the STRING database (http://string-db.org/ (accessed on 1 September 2022)), the protein–protein interaction network of Sdc-1 and TF pathway-related genes with respect to IL6, IL8, VEGF, and IGF-1R was determined via STRING analysis. (**A**) Interaction network of Sdc-1 (violet box), TF-pathway-related genes (TF, F7, F2R, F2RL1, and TFPI) (blue box) and IL6, IL8, VEGF, and IGF-1R (red boxes). Gene ontology (GO) analysis: (**B**) KEGG pathway analysis, (**C**) biological process, (**D**) molecular function, and (**E**) cellular components. The graphs for biological process (gray), molecular functions (yellow), and cellular components (violet), and the pie chart for KEGG pathways revealed the ten most significant GO keywords (*p* < 0.05). The log10 transformation was used to present the top 10 values. Tables S4–S6 contain further information.

The capability of Sdc-1 to act as a coreceptor and to interact with various growth factors, cytokines, chemokines, and adhesion molecules including VEGF, integrins, Wnt, IL6/JAK-STAT3, NFkB, and others is one of its most significant features [2]. We explored these protein interactions in silico using the STRING database to display the interaction network between Sdc-1 and factors involved in the TF pathway and downstream signaling pathways [21]. Figure 6A–D and Table S4 show the biological processes, molecular functions, and cellular components of the analyzed proteins. Interestingly, we identified that Sdc-1 is directly linked to VEGF, IL6, IL8, and IGF-1R, which are linked to TF pathway targets. This can suggest an indirect impact of Sdc-1 on the TF pathway. We also identified enriched pathways linked to cancer, PI3K-AKT, Rap1, Ras, EGFR, HIF-1, MAPK, and phospholipase signaling pathways (Figure 6E and Table S5). Based on PubMed co-citation analysis, Table S6 lists the top 10 considerably enriched phrases, which include a number of terms related to cancer.

## 4. Discussion

In this study, we intended to elucidate the cellular and functional relationships between the TF pathway and Sdc-1. Based on previous findings showing that Sdc-1-KD negatively affected angiogenesis in a TF pathway-dependent manner in a breast cancer cell–endothelial cell co-culture model [11], we present here, to the best of our knowledge, the first functional link between Sdc-1 and different components of the TF pathway in the context of breast cancer progression.

We have shown that various cytokines and pathway inhibitors are directly linked to Sdc-1 and TF function. Apart from the confirmation of the dysregulation of several TF pathway constituents in Sdc-1-depleted MDA-MB-231 cells [11], we noted that treatment of control cells with IL6, IL8, and IGFR inhibitors resulted in the differential regulation of various TF pathway constituents at the transcriptional level. In addition, a modest effect of TFPI treatment on TF expression was observed. Notably, the significant downregulation of TF and F7 by IL6 and VEGF, of F2R by IL6 and IL8, and of TF by IGFR inhibition in control cells were abolished or reverted under Sdc-1 KD conditions, indicating the Sdc-1-dependence of TF pathway expression for these cytokines. Indeed, this effect conforms to the classical role of Sdc-1 as a co-receptor for cytokine signaling [2]. The Sdc-1 extracellular domain serves as a potent binding site for numerous factors, including cytokines [1,6]. For example, IL6 displays multiple predicted heparin-binding sites and thus interactions with extracellular HS chains are thought to accumulate IL6 in proximity to the corresponding cell membrane, rendering IL6 more resistant to proteolytic degradation [26]. Several studies have provided evidence for regulatory feedback loops between Sdc-1 and IL6. For instance, IL6 modulated Sdc-1 expression in a variety of cells [6], whereas IL6 expression was found to be dysregulated in Sdc-1-depleted endometriotic cells [27] and Sdc-1-deficient mice [28]. Moreover, Sdc-1 directly mediates IL6-dependent effects on breast cancer cell adhesion and migration [29]. In our study, Sdc-1-KD may have decreased the potential binding sites for IL6 at the cell membrane and therefore diminished its effect on the expression of certain TF pathway components. Both IL6 and IL8 affect TF expression levels, although in monocytes a rather contradictory effect has been observed, compared to our results [30]. Additionally, in vivo evidence suggests significantly reduced IL6 and IL8 levels after inhibition of the TF/FVII complex. This functional link is strengthened by findings that IL6 treatment restores the reduced migration of Sdc-1-depleted MDA-MB-231 cells to the wild-type level. A link between IL6, IL8, Sdc-1, and the TF pathway was also evident in our downstream analysis, where TFPI treatment reduced IL6 expression and Sdc-1-KD reduced IL8 expression, respectively.

The postulated impact of Sdc-1 on signaling was confirmed by our phosphokinase activation analysis, where we observed a reduction in MAPK1 activation upon Sdc-1-KD. A striking finding was the synergistic activation of PTEN, MAPK, and STAT3 upon combined Sdc-1-KD and TFPI treatment. Although we can currently only speculate on the detailed mechanism underlying this process, this finding provides further evidence for the functional interaction of these pathways. This interaction was also confirmed by STRING analysis, suggesting an indirect connection between Sdc-1 function and the TF pathway via IL8 and VEGF.

Generally, the TF/FVIIa module transactivates RTKs in several contexts [31], and its role in mediating cell survival signals is well-established [32]. Aberg et al. found that the TF/FVIIa-mediated transactivation of IGF-1R protected, e.g., MDA-MB-231 breast cancer cells from apoptosis [33]. This effect was postulated to be dependent on the proximity of TFs and IGF-1R proteins at the plasma membrane and FVIIa proteolytic activity, thus being independent of downstream thrombin production and signaling via the TF cytoplasmic domain.

Accordingly, the decreased IGF-1R protein levels observed following TFPI application in the present study might have occurred due to decreased TF/FVIIa activity and thus less transactivation of IGF-1R, which in turn may be internalized to a greater extent in its inactivated form to be proteolytically degraded. Since IGF-1R has been described as an anti-apoptotic factor in breast cancer cells, increased apoptosis observed in TFPI-stimulated MDA-MB-231 cells may be a consequence of diminished IGF-1R levels as a result of TF pathway inhibition. Additional Sdc-1 depletion may subtract TFPI binding sites at the cell surface and thus reduce its inhibitory activity on the TF pathway.

At the functional level, we observed differential effects of Sdc-1-depletion on cell motility, cell viability, cell cycle progression, and apoptosis. In accordance with previous observations [4], Sdc-1-depleted MDA-MB-231 cells showed increased cell motility in migration assays. However, we did not observe an impact of TFPI treatment in these assays, indicating that the Sdc-1-dependence of cell motility is not linked to TF pathway inhibition. In contrast, TFPI treatment of control cells reduced cell viability and enhanced the G2/M-phase of the cell cycle. Both effects were abolished by Sdc-1-depletion, providing a functional link between both pathways. Generally, TFPI serves as an endogenous inhibitor of TF-induced blood coagulation by binding the active sites of the TF/FVIIa tandem and FXa [34]. Several studies have suggested an association between the soluble TFPIα isoform and different cell membrane HSPGs [35,36].

Moreover, TFPI treatment enhanced apoptosis in control cells, and this effect was not observed in Sdc-1-depleted cells. Indeed, Sdc-1-depletion in MDA-MB-231 cells under low serum conditions exerts an anti-apoptotic effect [17], and similar mechanisms may have been active in our current study. Generally, TFPI expression or treatment has been shown to be related to apoptosis in a regulatory manner in different cellular contexts [37,38]. The overexpression of TFPI or the treatment of cells with its recombinant form induced apoptotic cell death in cancer cells and thus a tumor-suppressing function was attributed to the protein. These findings may provide an explanation for the increased apoptosis, as well as the decreased proliferation, suggested by the significant G2/M phase cell-cycle arrest observed in TFPI-treated breast cancer cells. However, the notion that Sdc-1 in particular acts as a potent binding site for human rTFPI to facilitate its activity in a cell biological context remains to be evidenced.

In accordance with previous findings [9], Sdc-1 showed variable expression in different breast cancer subtypes. Notably, in both MCF-7 and MDA-MB-231 cells, representing different subtypes and degrees of aggressiveness of breast cancer, Sdc-1 overexpression was clearly associated with increased TF expression, which confirms the Sdc-1/TF axis at the protein level. TF expression was demonstrated to predict a poor prognosis in breast cancer [11,39] and to correlate with the grade of malignancy in glioma and pancreatic cancer [40]. This relation can be explained by considering the role of TF in inducing the coagulation cascade by binding FVII at the tumor cell surface. The subsequent local formation of thrombin can activate endothelial cells and platelets in the tumor microenvironment, thus triggering the formation of a metastatic niche. Here, we reflect on these findings in light of the indicated Sdc-1/TF axis, showing that the Sdc-1 overexpressing cells indeed display a higher potential to generate thrombin and thus to activate platelets more strongly. Although our data were restricted to two cell lines, this is a clear indicator of a new understanding of the relationship between Sdc-1 expression and coagulation via the TF axis.

Some limitations are associated with our study. Our study was designed as an in vitro study on cell lines, which are widely used models that are representative of basal and luminal breast cancer, but which may not be representative of the individual differences observed in primary cancer cells. Moreover, with the exception of functional breast-cancer–platelet interactions, the interplay of the Sdc-1-TF pathway with additional cell types of the tumor microenvironment was not evaluated in our experimental setup. As mentioned, the focus on MDA-MB-231 cells allowed for a more detailed study, and the use of consistent data in the overexpression and knockdown approaches increase our level of confidence in our data. Nevertheless, cell-type-specific effects of Sdc-1 on the TF pathway cannot be excluded, and these have partially been observed in a previous study with a focus on Sdc-1-dependent tumor angiogenesis [11]. Context-dependent effects of Sdc-1 are well-documented, as it is a multifunctional regulator of cell signaling [2]. Another limitation of our study is the investigation of several factors at the transcriptional level, which was confirmed at the protein level for tissue factors only. The reader is referred to our previous study [11] for the demonstration of Sdc-1-dependent regulation at the secretome level in an angiogenesis-coculture setting. Further studies in more complex experimental systems involving the tumor microenvironment are necessary in order to assess the full impact of our novel findings.

## 5. Conclusions

We concluded that the functional interplay of Sdc-1 and the TF pathway is compatible with the classical co-receptor role of Sdc-1 for cytokine signaling. Cell viability, migration apoptosis, and cell-cycle progression were affected by this regulatory axis. Our study opens a new functional avenue of research related to the notion that Sdc-1 fosters coagulation and platelet communication as a key process in the hematogenous metastatic spread of breast cancer cells.

## Data Availability

The data presented in this study are available in the article and Supplementary Material.

## Supplementary Materials

The following supporting information can be downloaded at: https://www.mdpi.com/article/10.3390/cells12060910/s1, Figure S1: qPCR analysis of gene expression changes in the breast cancer cells used in this study. Table S1: Different concentrations used for treatment of MDA-MB-231 cells. Table S2: PCR primers used in this study. Table S3: Antibodies used in this study. Table S4: GO enrichment analysis associated with TF targets (F3, F7, F2R, F2RL1, and TFPI) and Sdc-1 with respect to IL6, IL8, VEGF, and IGF-IR. Table S5: KEGG pathways associated with TF targets (F3, F7, F2R, F2RL1, and TFPI) and Sdc-1 with respect to IL6, IL8, VEGF, and IGF-IR according to KEGG enrichment analysis. Table S6: Statistical co-citation analysis associated with TF targets (F3, F7, F2R, F2RL1, and TFPI) and Sdc-1 with respect to IL6, IL8, VEGF, and IGF-IR according to PubMed.
