# Supplementary material for: The Heparan Sulfate Proteoglycan Syndecan-1 Triggers Breast Cancer Cell-Induced Coagulability by Induced Expression of Tissue Factor
Source: Cells. 2023 Mar 16;12(6):910. doi: 10.3390/cells12060910 (PMC10047229; doi:10.3390/cells12060910)
Supplement: Supplementary file 1 [file cells-12-00910-s001.zip › Supplementary figure_neu.pdf]

Supplementary Figure

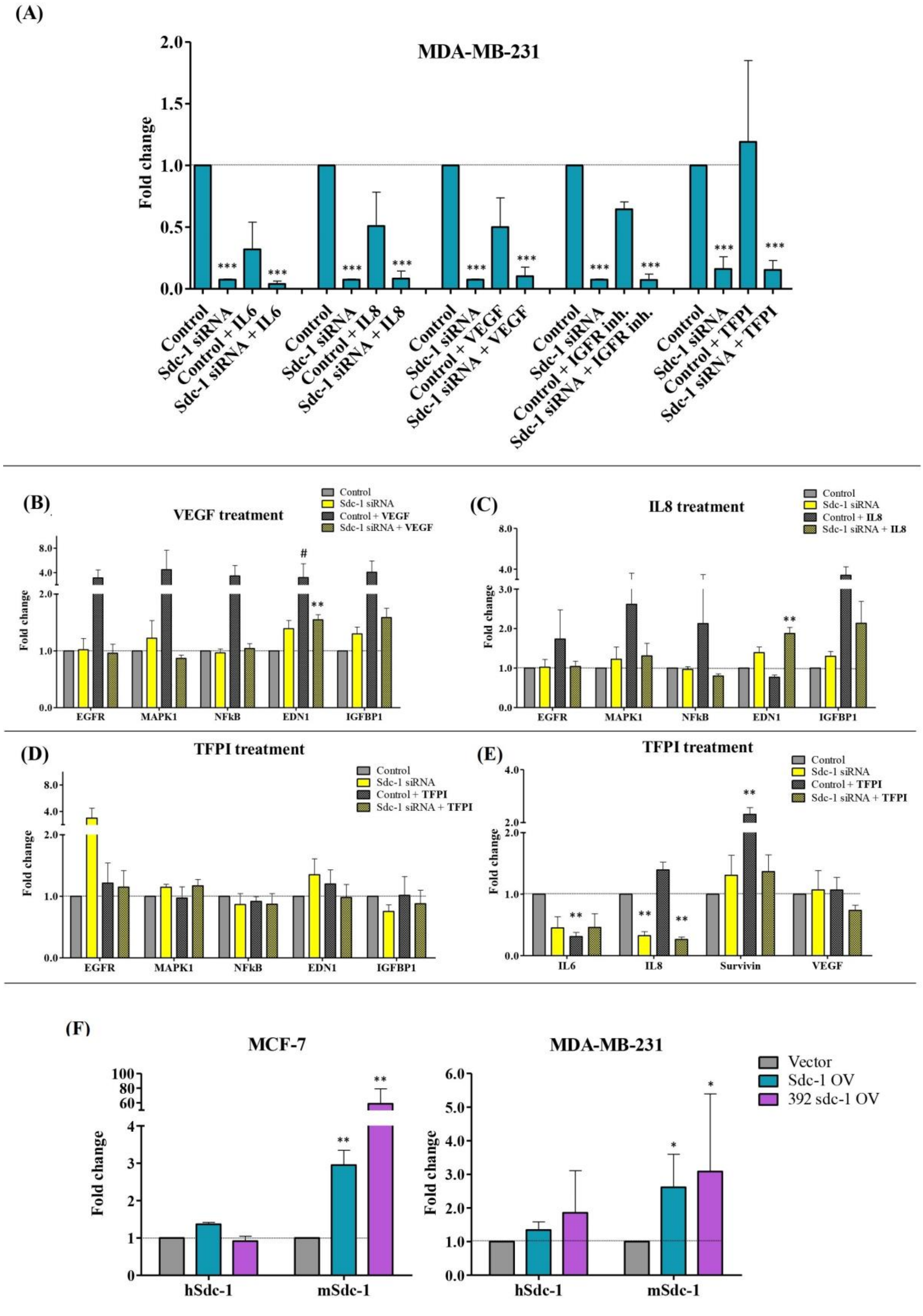

Supplementary Figure S1. qPCR analysis of gene expression changes in the breast cancer cells used in this study. (A) Sdc-1 siRNA transfection was confirmed by estimating the expression of Sdc-1 as determined by quantitative PCR after 72 h post-transfection and 48 h post-treatment with exogenous IL6 (100 ng/mL), IL8 (100 ng/mL), VEGF (20 ng/mL), TFPI (50 ng/mL) and the inhibition of IGFR with AG-1024 (20 μM), normalized to β-ACTIN expression and plotted as fold change. (B–E) The expression of different genes was analyzed post-transfection and 48 h after (B) VEGF (20 ng/mL), (C) IL8 (100 ng/mL), and (D,E) 50 μg/mL TFPI treatment via qPCR, normalized to β-actin expression, and shown as fold change (n = 3). Data represent the mean ± SEM. Asterisks on the top of the bars indicate significant differences between the controls and all the conditions. The lines over the bars indicate significant differences (* $p < 0.05$, ** $p < 0.01$, *** $p < 0.001$). (F) qPCR analysis of murine (mSdc-1) and human (hSdc-1) Sdc-1 expression. Sdc-1 expression was normalized to 18S rRNA. Error bars = SEM. Data represent the mean ± SEM from three independent experiments. Asterisks represent comparisons with statistically significant differences (# $p < 0.08$, * $p < 0.05$, ** $p < 0.01$, *** $p < 0.001$).
