# Supplementary material for: The Heparan Sulfate Proteoglycan Syndecan-1 Triggers Breast Cancer Cell-Induced Coagulability by Induced Expression of Tissue Factor
Source: Cells. 2023 Mar 16;12(6):910. doi: 10.3390/cells12060910 (PMC10047229; doi:10.3390/cells12060910)
Supplement: Supplementary file 1 [file cells-12-00910-s001.zip › Supplementary tables_neu.pdf]

**Supplementary Table 1. Different concentrations used for stimulation of MDA-MB-231 cells.**

| **Stimulant/inhibitor** | **Applied concentration** | **Cat. no.** | **Supplier** |
|---|---|---|---|
| **IL6** | 100 ng/ml | 11340064 | ImmunoTools, Friesoythe, Germany |
| **IL8** | 100 ng/ml | 11349084 | ImmunoTools, Friesoythe, Germany |
| **VEGF** | 20 ng/ml | ACFP293 | R&D Systems, Minnesota, United States |
| **IGFR inhibitor (AG-1024)** | 20 µM | S1234 | Selleck, Berlin, Germany |
| **TFPI** | 50 ng/ml | SRP6458 | Merck KGaA, Darmstadt, Germany |

**Supplementary Table 2. PCR primers used in this study.**

| **Gene** | **Forward primer sequences** | **Reverse primer sequences** |
|---|---|---|
| **β-Actin** | 5’-CAA AGA CCT GTA CGC CAA CAC-3’ | 5’-CAT ACT CCT GCT TGC TGA TCC-3’ |
| **Sdc-1** | 5’-AGG ACG AAG GCA GCT ACT CCT-3’ | 5’-TTT GGT GGG CTT CTG GTA GG-3’ |
| **F3** | 5’-CAG AGT TCA CAC CTT ACC TGG AG-3’ | 5’-GTT GTT CCT TCT GAC TAA AGT CCG-3’ |
| **F7** | 5’-CCT CAA GTC CAT GCC AGA ATG G-3’ | 5’-CAC AGA TCA GCT GGT CAT CCT TG-3’ |
| **F2R** | 5’-GTT TCT GGC TGT GGT GTA TCC C-3’ | 5’-CCT GGA TGG TTT GCT CCT TGA G-3’ |
| **F2RL1** | 5’-CTC CTC TCT GTC ATC TGG TTC C-3’ | 5’-TGC ACA CTG AGG CAG GTC ATG A-3’ |
| **EGFR** | 5’-CAATTGGAAGATTGGAAGATTCAGC-3’ | 5’-CCAGTCAGGTTACAGGGCACA-3’ |
| **MAPK1** | 5’-ACACCAACCTCTCGTACATCGG-3’ | 5’-TGGCAGTAGGTCTGGTGCTCAA-3’ |
| **NFkB** | 5’-GCAGCACTACTTCTTGACCACC-3’ | 5’-TCTGCTCCTGAGCATTGACGTC-3’ |
| **EDN1** | 5’-CTACTTCTGCCACCTGGACATC-3’ | 5’-TCACGGTCTGTTGCCTTTGTGG-3’ |
| **VEGFa** | 5’-TTGCCTTGCTGCTCTACCTCCA-3’ | 5’-GATGGCAGTAGCTGCGCTGATA-3’ |
| **IGFBP1** | 5’-TCCTTTGGGACGCCATCAGTAC-3’ | 5’- GATGTCTCCTGTGCCTTGGCTA-3’ |
| **IL6** | 5’-AGTGAGGAACAAGCCAGAGC-3’ | 5’-CATTTGTGGTTGGGTCAGG-3’ |

| | | |
|---|---|---|
| **IL8** | 5'-GAGAGTGATTGAGAGTGGACCAC-3' | 5'-CACAACCCTCTGCACCCAGTTT-3' |
| **Survivin** | 5'-CCACTGAGAACGAGCCAGACTT-3' | 5'-GTATTACAGGCGTAAGCCACCG-3' |

**Supplementary Table 3. Antibodies used in this study.**

| **Antibody** | **Cat. no.** | **Supplier** |
|---|---|---|
| p-PTEN (Ser380) | 9551 | Cell Signaling Technology, Massachusetts, United States |
| p-p44/42 MAPK (Erk1/2) (Thr202/Tyr204) | 9101 | Cell Signaling Technology, Massachusetts, United States |
| p-STAT3 (Tyr705) | 9131s | Cell Signaling Technology, Massachusetts, United States |
| IGF-I Receptor β | 3027 | Cell Signaling Technology, Massachusetts, United States |
| p-NFkB p65 (Ser536) | 3033s | Cell Signaling Technology, Massachusetts, United States |
| GAPDH | sc-47724 | Santa Cruz Biotechnology, Texas, United States |
| Goat Anti-Rabbit IgG | 401353 | Merck KGaA, Darmstadt, Germany |
| Goat Anti-Mouse IgG | 401253 | Merck KGaA, Darmstadt, Germany |

**Supplementary Table 4. GO enrichment analysis associated with TF targets (F3, F7, F2R, F2RL1, and TFPI), Sdc-1 with respect to IL6, IL8, VEGF and IGF-IR.** The program STRING was used for this analysis.

| **Category** | **Pathway description** | **Observed gene count** | **False discovery rate** | **Matching proteins in your network (labels)** |
|---|---|---|---|---|
| **Molecular function** | Vascular endothelial growth factor-activated receptor activity | 4 | 8,40E-07 | FLT4,KDR,NRP1,FLT1 |
| | Vascular endothelial growth factor binding | 4 | 8,40E-07 | FLT4,KDR,NRP1,FLT1 |
| | Transmembrane receptor protein tyrosine kinase activity | 5 | 5,90E-06 | FLT4,KDR,NRP1,IGF1R,FLT1 |
| | Signaling receptor binding | 12 | 5,90E-06 | SDCBP,KDR,IGF1R,F2RL1,IGF1,IRS1,CXCL8,F2R,PTPN11,F7,IL6,VEGFA |

| | | | | |
|---|---|---|---|---|
| | Insulin receptor binding | 4 | 1,03E-05 | IGF1R,IGF1,IRS1,PTPN11 |
| | Protein binding | 19 | 2,19E-05 | SDCBP,FLT4,KDR,NRP1,IGF1R,FLT1,CXCR1,F2RL1,IGF1,IRS1,CXCL8,CXCR2,F2R,F3,PTPN11,F7,SDC1,IL6,VEGFA |
| | Transmembrane signaling receptor activity | 10 | 4,48E-05 | FLT4,KDR,NRP1,IGF1R,FLT1,CXCR1,F2RL1,CXCR2,F2R,F3 |
| | Growth factor binding | 5 | 9,20E-05 | FLT4,KDR,NRP1,IGF1R,FLT1 |
| | Interleukin-8 receptor activity | 2 | 0,0015 | CXCR1,CXCR2 |
| | Interleukin-8 binding | 2 | 0,0023 | CXCR1,CXCR2 |
| **Cellular component** | Intrinsic component of plasma membrane | 13 | 1,26E-06 | SDCBP,FLT4,KDR,NRP1,IGF1R,FLT1,F2RL1,IRS1,CXCR2,F2R,F3,SDC1,IL6 |
| | Receptor complex | 8 | 1,96E-06 | SDCBP,FLT4,KDR,NRP1,IGF1R,FLT1,IRS1,IL6 |
| | Integral component of plasma membrane | 12 | 4,17E-06 | SDCBP,FLT4,KDR,NRP1,IGF1R,FLT1,F2RL1,IRS1,CXCR2,F2R,SDC1,IL6 |
| | Plasma membrane | 17 | 4,83E-05 | TFPI,SDCBP,FLT4,KDR,NRP1,IGF1R,FLT1,CXCR1,F2RL1,IGF1,IRS1,CXCR2,F2R,F3,F7,SDC1,IL6 |
| | Cell surface | 8 | 0,00023 | TFPI,NRP1,CXCR1,CXCR2,F2R,F3,SDC1,VEGFA |
| | Extracellular region | 14 | 0,001 | TFPI,SDCBP,FLT4,KDR,NRP1,FLT1,IGF1,CXCL8,F2R,F3,F7,SDC1,IL6,VEGFA |
| | Serine-type peptidase complex | 2 | 0,0021 | F3,F7 |
| | Insulin receptor complex | 2 | 0,0023 | IGF1R,IRS1 |
| | Endomembrane system | 14 | 0,0023 | TFPI,SDCBP,KDR,NRP1,FLT1,CXCR1,F2RL1,IGF1,CXCR2,F2R,F7,SDC1,IL6,VEGFA |
| | Intrinsic component of membrane | 15 | 0,0023 | TFPI,SDCBP,FLT4,KDR,NRP1,IGF1R,FLT1,CXCR1,F2RL1,IRS1,CXCR2,F2R,F3,SDC1,IL6 |
| **Biological process** | Positive regulation of cell migration | 15 | 5,39E-16 | SDCBP,FLT4,KDR,NRP1,IGF1R,FLT1,F2RL1,IGF1,CXCL8,CXCR2,F2R,F3,F7,IL6,VEGFA |
| | Positive regulation of intracellular signal transduction | 15 | 1,47E-12 | SDCBP,FLT4,KDR,NRP1,IGF1R,FLT1,F2RL1,IGF1,IRS1,F2R,F3,PTPN11,F7,IL6,VEGFA |
| | Positive regulation of chemotaxis | 9 | 2,05E-11 | KDR,NRP1,F2RL1,CXCL8,CXCR2,F3,F7,IL6,VEGFA |
| | Positive regulation of mapk | 12 | 2,85E-11 | SDCBP,FLT4,KDR,NRP1,IGF1R,FLT1,F2RL1,IGF1,F2R,PTPN11,IL6,VEGFA |

| | | | | |
|---|---|---|---|---|
| | cascade | | | |
| | Regulation of localization | 18 | 6,91E-11 | SDCBP,FLT4,KDR,NRP1,IGF1R,FLT1,F2RL1,IGF1,IRS1,CXCL8,CXCR2,F2R,F3,PTPN11,F7,SDC1,IL6,VEGFA |
| | Positive regulation of multicellular organismal process | 16 | 7,21E-11 | SDCBP,FLT4,KDR,NRP1,IGF1R,FLT1,F2RL1,IGF1,CXCL8,CXCR2,F2R,F3,PTPN11,F7,IL6,VEGFA |
| | Positive regulation of response to stimulus | 17 | 8,89E-11 | SDCBP,FLT4,KDR,NRP1,IGF1R,FLT1,F2RL1,IGF1,IRS1,CXCL8,CXCR2,F2R,F3,PTPN11,F7,IL6,VEGFA |
| | Positive regulation of cell population proliferation | 13 | 2,88E-10 | SDCBP,FLT4,KDR,NRP1,IGF1R,FLT1,IGF1,IRS1,CXCR2,F2R,F3,IL6,VEGFA |
| | Response to organic substance | 18 | 2,88E-10 | TFPI,FLT4,KDR,NRP1,IGF1R,FLT1,CXCR1,IGF1,IRS1,CXCL8,CXCR2,F2R,F3,PTPN11,F7,SDC1,IL6,VEGFA |
| | Positive regulation of positive chemotaxis | 6 | 3,33E-10 | KDR,F2RL1,CXCL8,F3,F7,VEGFA |

**Supplementary Table 5. KEGG pathways associated with TF targets (F3, F7, F2R, F2RL1, and TFPI), Sdc-1 with respect to IL6, IL8, VEGF and IGF-IR according to KEEG enrichment analysis.** The program STRING was used for this analysis.

| Pathway description | Observed gene count | False discovery rate | Matching proteins in your network (labels) |
|---|---|---|---|
| PI3K-Akt signaling pathway | 9 | 9,98E-09 | FLT4,KDR,IGF1R,FLT1,IGF1,IRS1,F2R,IL6,VEGFA |
| Rap1 signaling pathway | 7 | 1,65E-07 | FLT4,KDR,IGF1R,FLT1,IGF1,F2R,VEGFA |
| Ras signaling pathway | 7 | 2,35E-07 | FLT4,KDR,IGF1R,FLT1,IGF1,PTPN11,VEGFA |
| EGFR tyrosine kinase inhibitor resistance | 5 | 1,50E-06 | KDR,IGF1R,IGF1,IL6,VEGFA |
| Focal adhesion | 6 | 2,58E-06 | FLT4,KDR,IGF1R,FLT1,IGF1,VEGFA |
| Proteoglycans in cancer | 6 | 2,58E-06 | KDR,IGF1R,IGF1,PTPN11,SDC1,VEGFA |
| HIF-1 signaling pathway | 5 | 3,71E-06 | IGF1R,FLT1,IGF1,IL6,VEGFA |
| MAPK signaling pathway | 6 | 1,48E-05 | FLT4,KDR,IGF1R,FLT1,IGF1,VEGFA |
| Phospholipase D signaling pathway | 5 | 1,48E-05 | CXCR1,CXCL8,CXCR2,F2R,PTPN11 |

| | | | |
|---|---|---|---|
| Pathways in cancer | 7 | 1,81E-05 | FLT4,IGF1R,IGF1,CXCL8,F2R,IL6,VEGFA |
| Epithelial cell signaling in Helicobacter pylori infection | 4 | 2,25E-05 | CXCR1,CXCL8,CXCR2,PTPN11 |
| Transcriptional misregulation in cancer | 5 | 2,25E-05 | IGF1R,FLT1,IGF1,CXCL8,IL6 |
| Complement and coagulation cascades | 4 | 4,11E-05 | TFPI,F2R,F3,F7 |
| Rheumatoid arthritis | 4 | 4,38E-05 | FLT1,CXCL8,IL6,VEGFA |

**Supplementary Table 6. Statistical co-citation analysis associated with TF targets (F3, F7, F2R, F2RL1, and TFPI), Sdc-1 with respect to IL6, IL8, VEGF and IGF-IR according to PubMed.** The program STRING was used for this analysis.

| Pathway description | Observed gene count | False discovery rate | Matching proteins in your network (labels) |
|---|---|---|---|
| (2011) Pharmacogenetic angiogenesis profiling for first-line Bevacizumab plus oxaliplatin-based chemotherapy in patients with metastatic colorectal cancer. | 9 | 2.27e-15 | KDR,NRP1,CXCR1,IGF1,CXCL8,CXCR2,F3,IL6,VEGFA |
| (2011) Targeting angiogenesis in childhood sarcomas. | 9 | 8.41e-14 | FLT4,KDR,IGF1R,FLT1,IGF1,IRS1,CXCL8,IL6,VEGFA |
| (2011) HCC and angiogenesis: possible targets and future directions. | 9 | 9.02e-14 | FLT4,KDR,FLT1,CXCR1,IGF1,CXCL8,CXCR2,IL6,VEGFA |
| (2016) The molecular mechanisms of hemodialysis vascular access failure. | 9 | 4.39e-13 | KDR,FLT1,IGF1,CXCL8,CXCR2,F3,SDC1,IL6,VEGFA |
| (2019) The role of vascular endothelial growth factor, interleukin 8, and insulin like growth factor in sustaining autophagic DIRAS3-induced dormant ovarian cancer xenografts. | 8 | 4.90e-13 | KDR,IGF1R,FLT1,CXCR1,IGF1,CXCL8,IL6,VEGFA |
| (2018) Receptor tyrosine kinases (RTKs) in breast cancer: signaling, therapeutic implications and challenges. | 10 | 8.42e-13 | FLT4,KDR,IGF1R,FLT1,IGF1,IRS1,CXCL8,SDC1,IL6,VEGF A |
| (2015) Modulation of cellular signaling by herpesvirus-encoded G protein-coupled receptors. | 10 | 8.42e-13 | KDR,IGF1R,FLT1,CXCR1,IGF1,CXCL8,CXCR2,F2R,IL6,VE GFA |
| (2013) Current status of bevacizumab in advanced ovarian cancer. | 8 | 8.42e-13 | FLT4,KDR,NRP1,FLT1,IGF1,CXCL8,IL6,VEGFA |
| (2016) Glioblastoma Stem Cells Microenvironment: The | 10 | 9.28e-13 | FLT4,KDR,IGF1R,FLT1,CXCR1,IGF1,CXCL8,CXCR2,IL6,V |

| | | | |
|---|---|---|---|
| Paracrine Roles of the Niche in Drug and Radioresistance. | | | EGFA |
| (2012) Integrin-mediated cell-matrix interaction in physiological and pathological blood vessel formation. | 10 | 1.23e-12 | TFPI,KDR,NRP1,FLT1,IRS1,CXCL8,F3,PTPN11,SDC1,VEGFA |
| (2011) Pharmacogenetic angiogenesis profiling for first-line Bevacizumab plus oxaliplatin-based chemotherapy in patients with metastatic colorectal cancer. | 9 | 2.27e-15 | KDR,NRP1,CXCR1,IGF1,CXCL8,CXCR2,F3,IL6,VEGFA |
| (2011) Targeting angiogenesis in childhood sarcomas. | 9 | 8.41e-14 | FLT4,KDR,IGF1R,FLT1,IGF1,IRS1,CXCL8,IL6,VEGFA |
| (2011) HCC and angiogenesis: possible targets and future directions. | 9 | 9.02e-14 | FLT4,KDR,FLT1,CXCR1,IGF1,CXCL8,CXCR2,IL6,VEGFA |
| (2016) The molecular mechanisms of hemodialysis vascular access failure. | 9 | 4.39e-13 | KDR,FLT1,IGF1,CXCL8,CXCR2,F3,SDC1,IL6,VEGFA |
| (2019) The role of vascular endothelial growth factor, interleukin 8, and insulin like growth factor in sustaining autophagic DIRAS3-induced dormant ovarian cancer xenografts. | 8 | 4.90e-13 | KDR,IGF1R,FLT1,CXCR1,IGF1,CXCL8,IL6,VEGFA |
